# Author Correction: Cytoplasmic LIF reprograms invasive mode to enhance NPC dissemination through modulating YAP1-FAK/PXN signaling

**DOI:** 10.1038/s41467-026-79112-5

**Published:** 2026-11-04

**Authors:** Shu-Chen Liu, Tien Hsu, Yu-Sun Chang, An-Ko Chung, Shih Sheng Jiang, Chun-Nan OuYang, Chiou-Hwa Yuh, Chuen Hsueh, Ya-Ping Liu, Ngan-Ming Tsang

**Affiliations:** 1 Department of Biomedical Sciences and Engineering, National Central University, 300, Zhongda Rd., Jhongli Dist., 32001 Taoyuan City, Taiwan; 2 Molecular Medicine Research Center, Chang Gung University, 259, Wenhua 1st Rd., Guishan Dist., 33302 Taoyuan City, Taiwan; 3 Graduate Institute of Biomedical Sciences, Chang Gung University, 259, Wenhua 1st Rd., Guishan Dist., 33302 Taoyuan City, Taiwan; 4 National Institute of Cancer Research, National Health Research Institutes, 35 Keyan Rd., Zhunan, 35053 Miaoli County, Taiwan; 5 Institute of Molecular and Genomic Medicine, National Health Research Institutes, 35 Keyan Rd., Zhunan, 35053 Miaoli County, Taiwan; 6 Department of Pathology, Chang Gung Memorial Hospital at Lin-Kou, 5 Fuxing St., Guishan Dist., 333 Taoyuan City, Taiwan; 7 Pathology Core of the Molecular Medicine Research Center, Chang Gung University, 5, Fuxing St., Guishan Dist., 333 Taoyuan City, Taiwan; 8 Department of Radiation Oncology, Chang Gung Memorial Hospital and University at Lin-Kou, 5, Fuxing St., Guishan Dist., 333 Taoyuan City, Taiwan

Correction to: *Nature Communications* https://doi.org/10.1038/s41467-018-07660-6, published online 30 November 2018

In the original version of this article, the right panel of Fig. 1c, showing recurrence-free survival according to LIF expression, was incorrectly labeled “LIFR Score.” The correct label is “LIF Score.” Figure 1c presents Kaplan–Meier analyses based on tumor LIF expression, whereas the corresponding survival analyses based on LIFR expression are shown separately in Fig. 1e. This labeling error does not affect the underlying data, statistical analyses, results, or conclusions of the article. Due to the age of the article, the figure cannot be updated directly. The revised figure is available as Supplementary Information accompanying this amendment. This notice serves to correct the article.

## Supplementary information

Corrected Fig. 1

