# Supplementary material for: Cytoplasmic LIF reprograms invasive mode to enhance NPC dissemination through modulating YAP1-FAK/PXN signaling
Source: Nat Commun. 2018 Nov 30;9:5105. doi: 10.1038/s41467-018-07660-6 (PMC6269507; doi:10.1038/s41467-018-07660-6)
Supplement: Supplementary file 1 — Supplementary Information [file 41467_2018_7660_MOESM1_ESM.pdf]

Supplementary Information

Cytoplasmic LIF reprograms invasive mode to enhance NPC dissemination through modulating YAP1-FAK/PXN signaling

Liu et al.

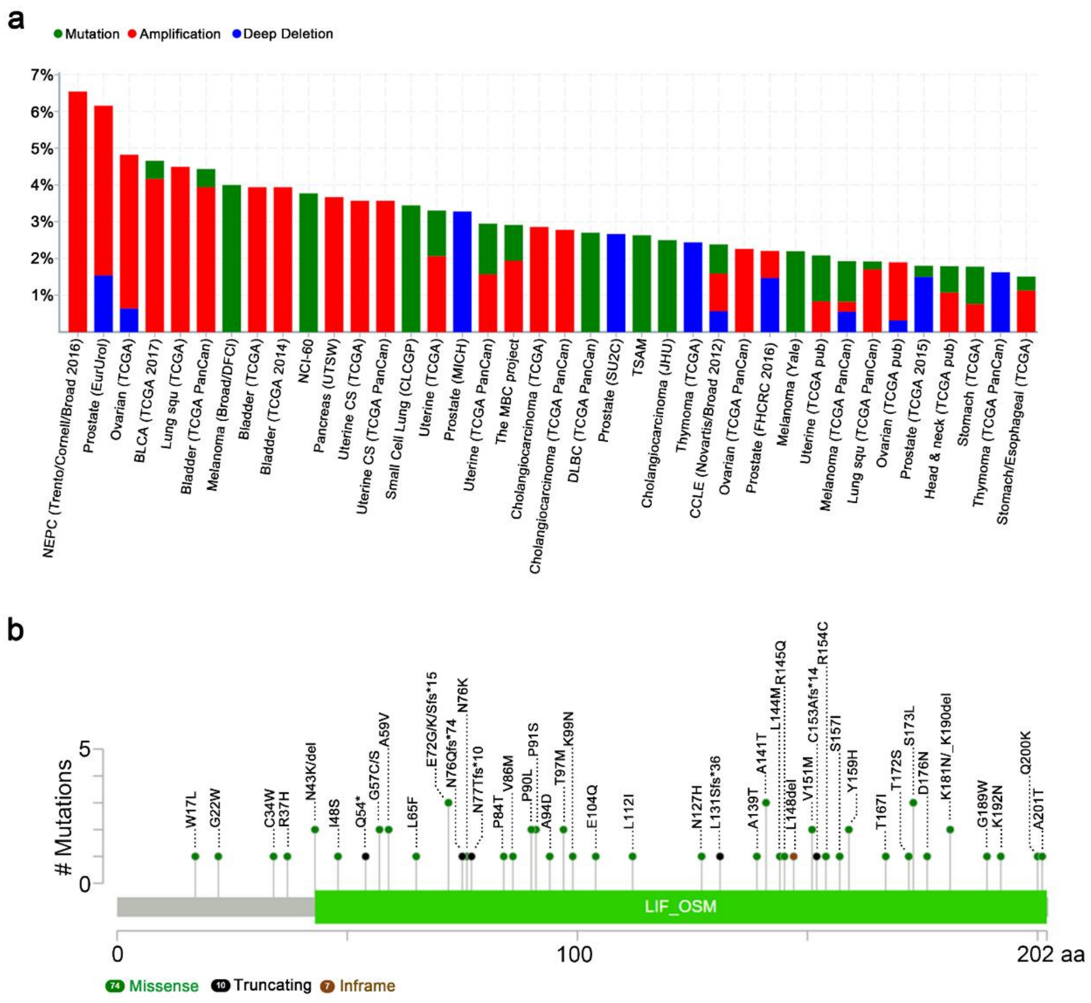

Supplementary Figure 1 Spectrum of LIF genetic alterations in cancers. **a** Summary of cross-cancer genetic alterations for LIF gene. **b** Diagram of LIF mutations. Circles are colored with respect to the corresponding mutation types. Data were extracted from cBioPortal database. A total of 216 studies were included in the analysis.

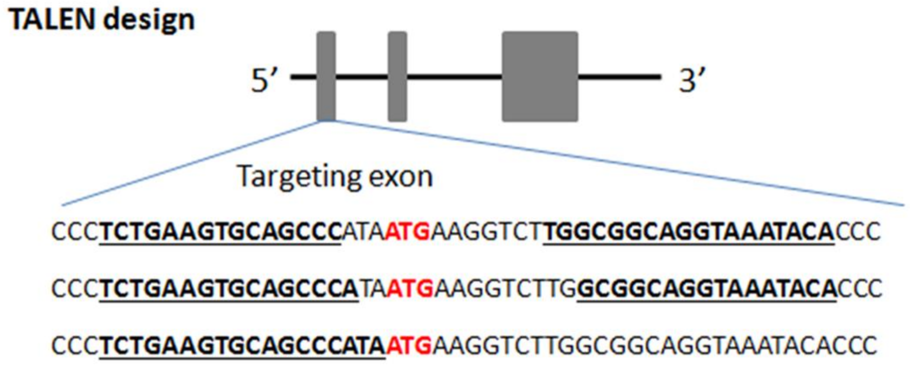

Supplementary Figure 2 Designation of TALEN constructs targeting to LIF.

TALEN binding sites in the LIF gene were shown. The underline indicated the left and right arms of TALEN targeting site. The initiating codon within the spacer was indicated in red.

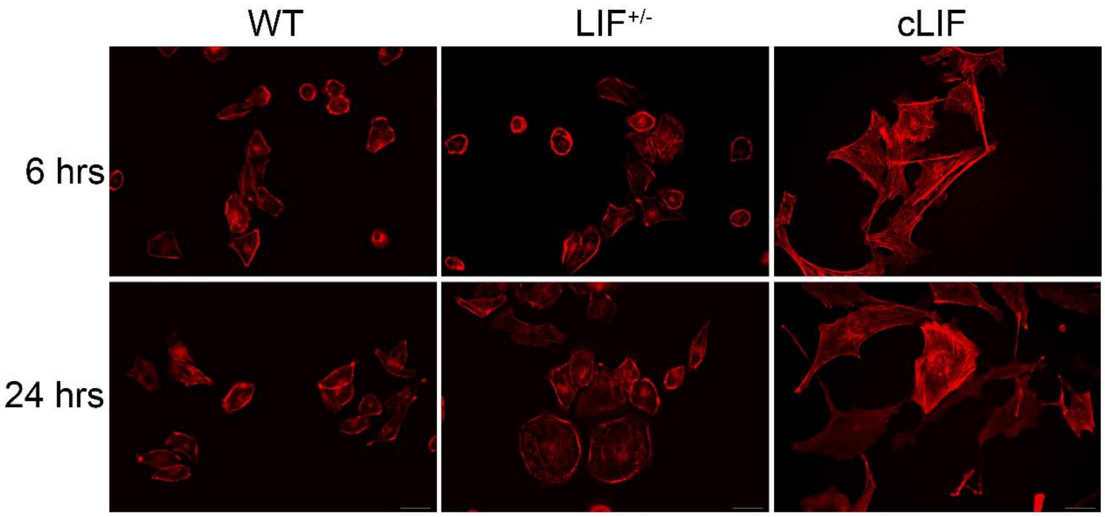

Supplementary Figure 3 Live-imaging analysis of cell attachment. Images of cancer cells expressing LifeAct-RFP were captured at 6 and 24 hours post plating. Scale bars, 20 μm. Shown are representative images of n = 3.

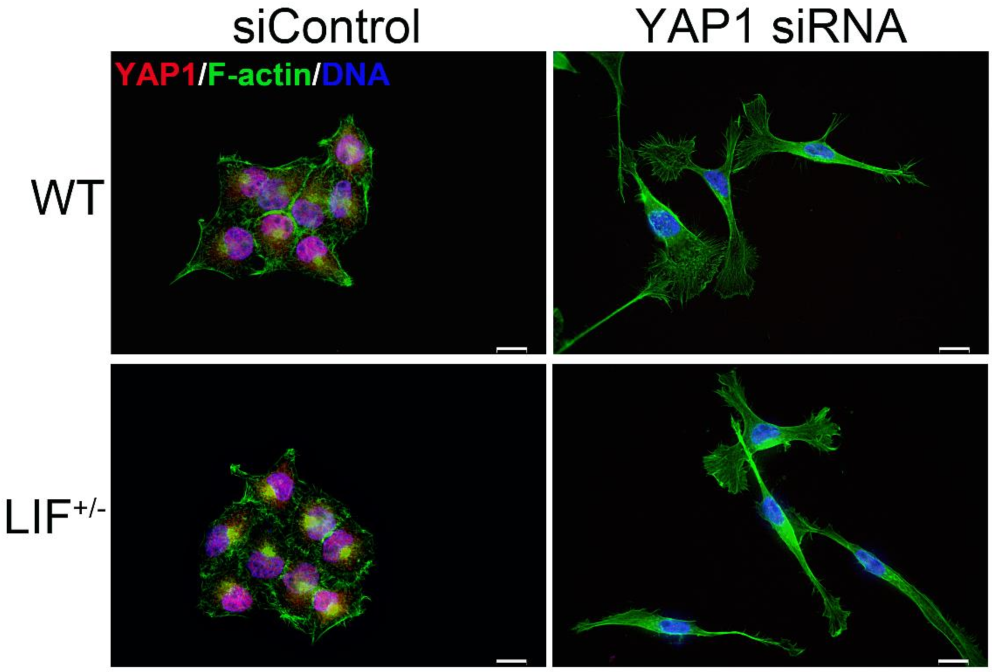

Supplementary Figure 4 Immunostaining for YAP1expressions in WT or LIF$^{+/-}$ cancer cells transfected with YAP1 siRNA or control siRNA. Alexa Fluor 488 phalloidin (green) was used to stain F-actin. Blue, DNA stained with Hoechst33342. Scale bars, 10 µm. Shown are representative images of n = 3.

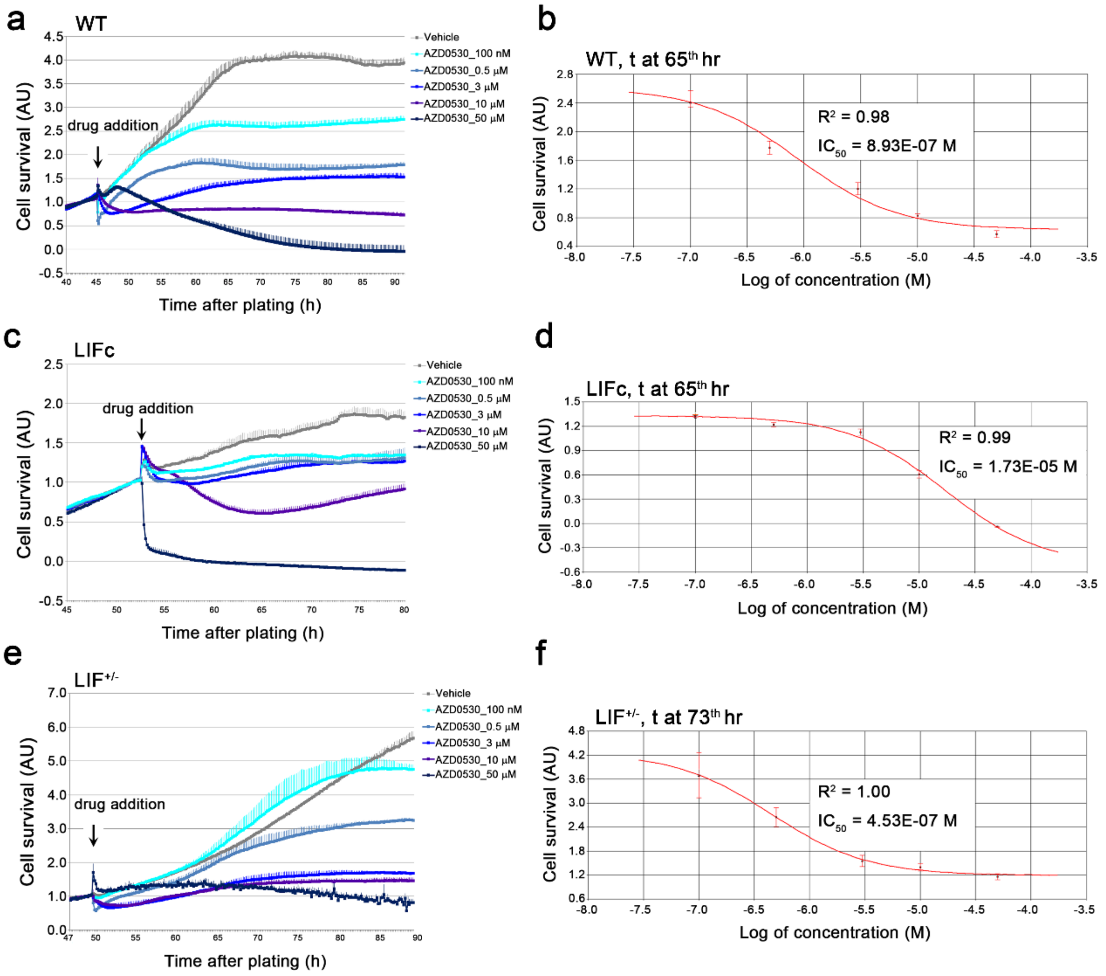

Supplementary Figure 5 Assessment of the IC_{50} values of AZD0530 for WT and LIF mutant cancer cells. **a, c, e** Real-time measurement of cell survival in response to various doses of AZD0530 treatment. Values of cell survival were normalized with respect to the time of AZD0530 addition. Values are presented as means and SD of quadruplicate experiments. **b, d, f** The AZD0530-induced cell toxicity was determined by calculating the IC_{50} after treatment. Normalized cell index was displayed against the logarithm of concentration. The values of IC_{50} were calculated using the RTCA software (ACEA Biosciences).

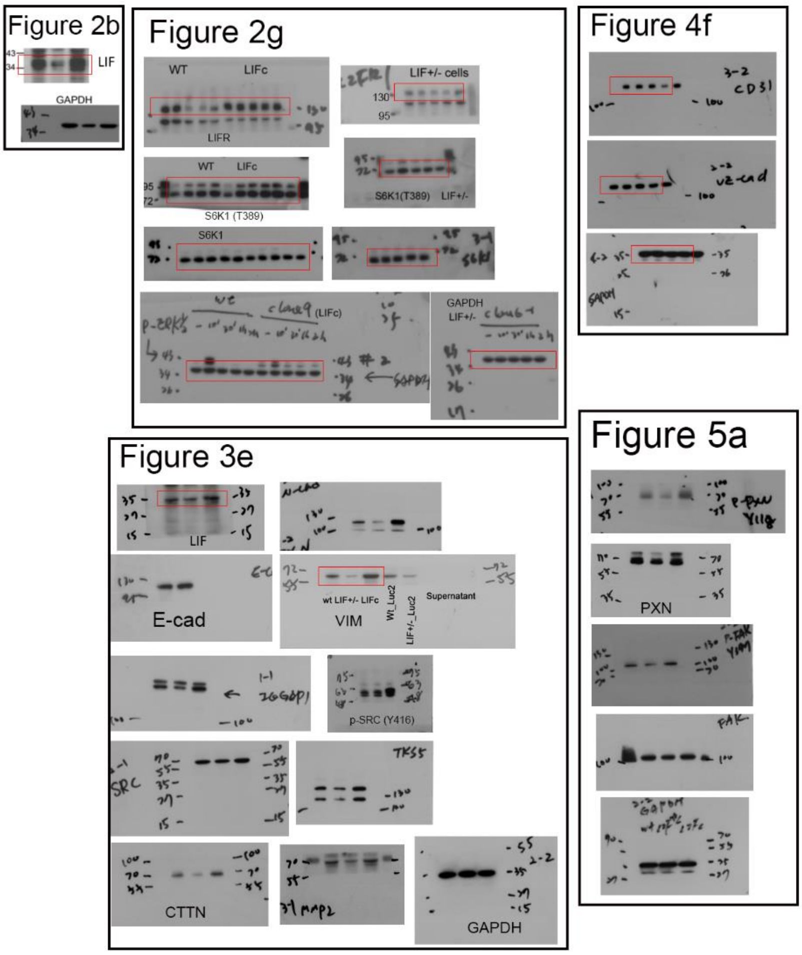

Supplementary Figure 6 Uncropped western blots from Figure 2-5.

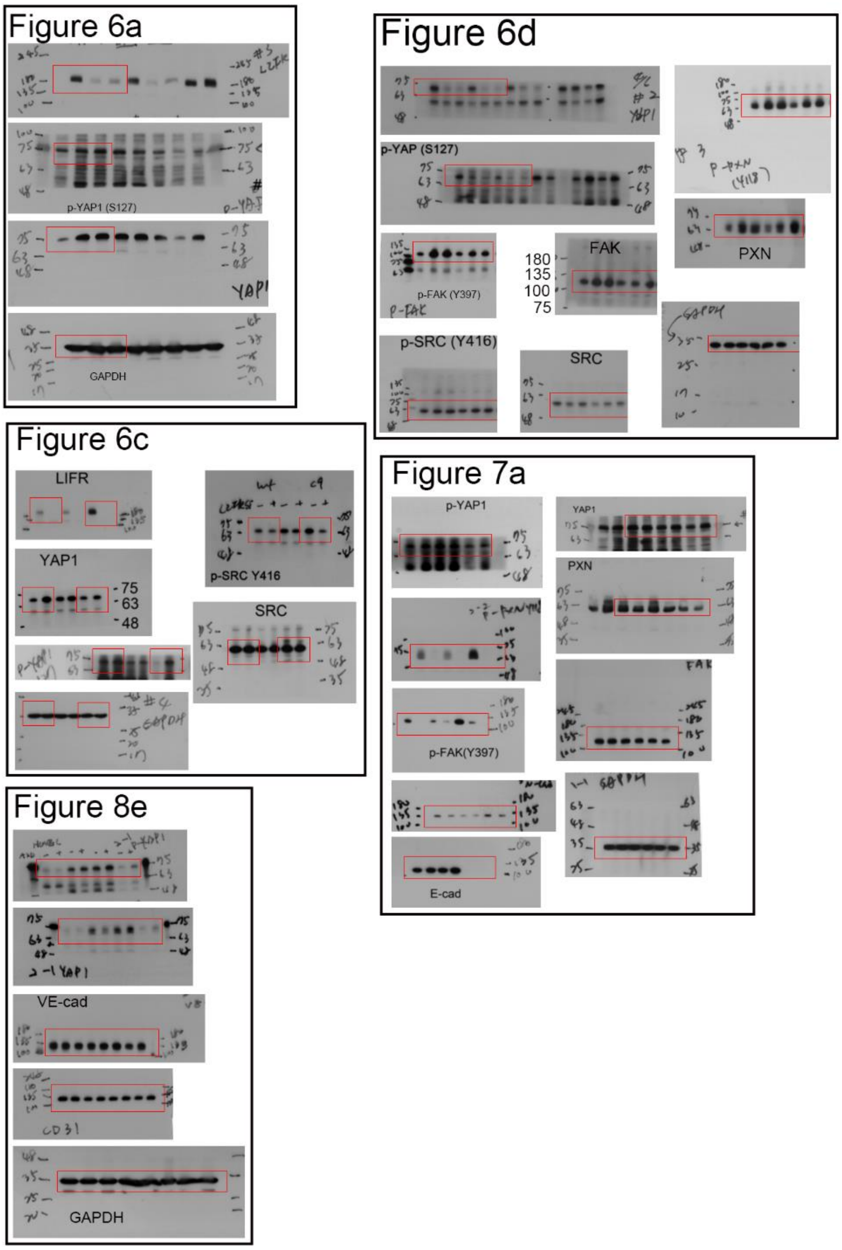

Supplementary Figure 7. Uncropped western blots from Figure 6-8.

Supplementary Table 1. Baseline Characteristics of the LIF Study Population

LIF Score	<40	≥40	Total	p value
Number	94 (50.8%)	91 (49.2%)	185 (100%)	
Age (years) Median	46.42 (25.58-80.70)	45.95 (22.48-78.94)	46.16 (22.48-80.70)	0.954[B]
Mean (±s.d.)	47.81±11.99	47.71±12.01	47.76±11.97	
Total Treatment Days	54.00 (45.00-68.00)	54.00 (44.00-70.00)	54.00 (44.00-70.00)	0.526[B]
Mean (±s.d.)	54.90±4.73	54.45±4.97	54.68±4.84	
Gender				0.866[A]
Female	24 (25.5%)	22 (24.2%)	46 (24.9%)	
Male	70 (74.5%)	69 (75.8%)	139 (75.1%)	
AJCC 2010 T-Stages				0.769[A]
$T_{2/1}$	53 (56.4%)	49 (53.8%)	102 (55.1%)	
$T_{4/3}$	41 (43.6%)	42 (46.2%)	83 (44.9%)	
AJCC 2010 N-Stage				0.769[A]
$N_{1/0}$	49 (52.1%)	50 (54.9%)	99 (53.5%)	
$N_{3/2}$	45 (47.9%)	41 (45.1%)	86 (46.5%)	
Chemotherapy				0.176[A]
No	8 (8.5%)	14 (15.4%)	22 (11.9%)	
Yes	86 (91.5%)	77 (84.6%)	163 (88.1%)	
Comorbidity				0.649[A]
No	62 (66.0%)	57 (62.6%)	119 (64.3%)	
Yes	32 (34.0%)	34 (37.4%)	66 (35.7%)	
Smoking				0.660[A]
No	49 (52.1%)	44 (48.4%)	93 (50.3%)	
Yes	45 (47.9%)	47 (51.6%)	92 (49.7%)	
Betel Quid				0.613[A]
No	72 (76.6%)	66 (72.5%)	138 (74.6%)	
Yes	22 (23.4%)	25 (27.5%)	47 (25.4%)	
Alcohol				0.069[A]
No	66 (70.2%)	52 (57.1%)	118 (63.8%)	
Yes	28 (29.8%)	39 (42.9%)	67 (36.2%)	
OS Medium years	7.64 (0.16-12.49)	6.57 (0.42-10.91)	7.13 (0.16-12.49)	0.003[B]
Mean (±s.d.)	7.08±2.89	5.82±2.91	6.46±2.96	

[A]obtained using chi-square test, 2-tailed. [B]p-value was calculated using ANOVA test, 2-tailed.

Supplementary Table 2. Univariate and Multivariate Analysis of LIF and Clinical Characteristics for Metastasis-Free Survival and Recurrence-Free Survival of NPC Patients

**Metastasis-Free Survival:**	Univariate Analysis		Multivariate Analysis	
n=185	HR (95% c.i.)	p value	HR (95% c.i.)	p value
LIF Score (≥40 vs. <40)	1.908 (1.027-3.544)	0.041	1.873 (1.007-3.482)	0.047
Treatment Days[A]	1.042 (0.982-1.105)	0.170		
Age[a]	1.009 (0.984-1.036)	0.470		
Gender (Male vs. Female)	1.540 (0.874-2.712)	0.135		
AJCC 2010 T-Stage ($T_{4/3}$ vs. $T_{2/1}$)	1.330 (0.731-2.419)	0.350		
AJCC 2010 N-Stage ($N_{3/2}$ vs. $N_{1/0}$)	2.037 (1.105-3.755)	0.023	2.037 (1.105-3.755)	0.023
Chemotherapy (Yes vs. no)	0.627 (0.279-1.409)	0.258		
Comorbidity (Yes vs. no)	1.272 (0.690-2.345)	0.440		
Smoking (Yes vs. no)	1.855 (1.000-3.444)	0.050	1.855 (1.000-3.444)	0.050
Betel Quid (Yes vs. no)	1.174 (0.603-2.287)	0.637		
Alcohol (Yes vs. no)	1.070 (0.576-1.986)	0.830		

**Recurrence-Free Survival:**	Univariate Analysis		Multivariate Analysis	
n=185	HR (95% c.i.)	p value	HR (95% c.i.)	p value
LIF Score (≥40 vs. <40)	1.885 (1.046-3.398)	0.035	1.838 (1.019-3.315)	0.043
Treatment Days[A]	1.026 (0.970-1.086)	0.373		
Age[a]	1.000 (0.976-1.025)	0.998		
Gender (Male vs. Female)	1.550 (0.750-3.207)	0.237		
AJCC 2010 T-Stage ($T_{4/3}$ vs. $T_{2/1}$)	1.812 (1.016-3.230)	0.044	1.764 (0.989-3.147)	0.055
AJCC 2010 N-Stage ($N_{3/2}$ vs. $N_{1/0}$)	1.131 (0.637-2.005)	0.675		
Chemotherapy (Yes vs. no)	1.085 (0.429-2.744)	0.864		
Comorbidity (Yes vs. no)	1.210 (0.672-2.180)	0.525		
Smoking (Yes vs. no)	1.671 (0.933-2.993)	0.084		
Betel Quid (Yes vs. no)	1.049 (0.544-2.021)	0.886		
Alcohol (Yes vs. no)	1.536 (0.864-2.730)	0.144		

[A]continuous variable

Supplementary Table 3. Baseline Characteristics of the LIFR Study Population

LIFR Score	<160	≥160	Total	p value
Number	96 (64.0%)	54 (36.0%)	150 (100%)	
Scoring (Median)	90.00 (0.00-150.0)	207.5 (160.0-270.0)	122.50(0.00-270.0)	< 0.001[B]
Mean (±s.d.)	89.84±37.22	212.87±41.58	134.13±70.77	
Age (years) Median	48.00 (26.10-77.71)	47.80 (26.00-81.35)	47.87 (26.00-81.35)	0.696[B]
Mean (±s.d.)	49.21±10.31	49.96±11.86	49.48±10.86	
Total Treatment Days	54.00 (44.00-79.00)	54.50 (45.00-78.00)	54.00 (44.00-79.00)	0.483[B]
Mean (±s.d.)	54.61±5.59	55.24±5.01	54.84±5.38	
Gender				0.790[A]
Female	25 (26.0%)	13 (24.1%)	38 (25.3%)	
Male	71 (74.0%)	41 (75.9%)	112 (74.7%)	
AJCC 2010 T-Stages				0.206[A]
$T_{2/1}$	53 (55.2%)	24 (44.4%)	77 (51.3%)	
$T_{4/3}$	43 (44.8%)	30 (55.6%)	73 (48.7%)	
AJCC 2010 N-Stage				0.461[A]
$N_{1/0}$	54(56.3%)	27 (50.0%)	81 (54.0%)	
$N_{3/2}$	42 (43.8%)	27 (50.0%)	69 (46.0%)	
Chemotherapy				0.217[A]
No	8 (8.3%)	8 (14.8%)	16(10.7%)	
Yes	88 (91.7%)	46 (85.2%)	134 (89.3%)	
Comorbidity				0.377[A]
No	57(59.4%)	36 (66.7%)	93 (62.0%)	
Yes	39 (40.6%)	18 (33.3%)	57 (38.0%)	
Smoking				0.558[A]
No	45 (46.9%)	28 (51.9%)	73 (48.7%)	
Yes	51 (53.1%)	26 (48.1%)	77 (51.3%)	
Betel Quid				0.757[A]
No	75(78.1%)	41(75.9%)	116 (77.3%)	
Yes	21 (21.9%)	13 (24.1%)	34 (22.7%)	
Alcohol				0.387[A]
No	59(61.5%)	37 (68.5%)	96 (64.0%)	
Yes	37 (38.5%)	17 (31.5%)	54 (36.0%)	
OS Medium years	7.85 (0.18-12.30)	6.28 (0.68-12.58)	7.50 (0.18-12.58)	0.057[B]
Mean (±s.d.)	7.66±3.12	6.58±3.60	7.27±3.33	

[A]obtained using chi-square test, 2-tailed. [B]p-value was calculated using ANOVA test, 2-tailed.

Supplementary Table 4. Univariate and Multivariate Analysis of LIFR and Clinical Characteristics for Metastasis-Free Survival and Recurrence-Free Survival of NPC Patients

**Metastasis-Free Survival:**	Univariate Analysis		Multivariate Analysis	
n=150	HR (95% c.i.)	p value	HR (95% c.i.)	p value
LIFR Score (≥ 160 vs. <160)	2.778 (1.359-5.679)	0.005	2.704 (1.322-5.532)	0.006
Treatment Days[A]	0.994 (0.962-1.028)	0.727		
Age[a]	1.031 (0.975-1.091)	0.286		
Gender (Male vs. Female)	2.471 (0.864-7.062)	0.091		
AJCC 2010 T-Stage ($T_{4/3}$ vs. $T_{2/1}$)	1.503 (0.737-3.069)	0.263		
AJCC 2010 N-Stage ($N_{3/2}$ vs. $N_{1/0}$)	2.356 (1.129-4.918)	0.022	2.280 (1.092-4.762)	0.028
Chemotherapy (Yes vs. no)	0.689 (0.241-1.970)	0.487		
Comorbidity (Yes vs. no)	0.961 (0.461-2.007)	0.916		
Smoking (Yes vs. no)	1.860 (0.891-3.881)	0.098		
Betel Quid (Yes vs. no)	1.060 (0.457-2.461)	0.892		
Alcohol (Yes vs. no)	1.164 (0.565-2.399)	0.68		

**Recurrence-Free Survival:**	Univariate Analysis		Multivariate Analysis	
n=150	HR (95% c.i.)	p value	HR (95% c.i.)	p value
LIFR Score (≥ 160 vs. <160)	2.305 (1.173-4.527)	0.015	2.198 (1.117-4.327)	0.023
Treatment Days[A]	0.989(0.957-1.021)	0.495		
Age[a]	1.048(0.997-1.101)	0.067		
Gender (Male vs. Female)	1.744 (0.722-4.213)	0.216		
AJCC 2010 T-Stage ($T_{4/3}$ vs. $T_{2/1}$)	2.060 (1.019-4.162)	0.044	1.949(0.963-3.945)	0.064
AJCC 2010 N-Stage ($N_{3/2}$ vs. $N_{1/0}$)	1.100 (0.561-2.158)	0.781		
Chemotherapy (Yes vs. no)	0.817 (0.288-2.323)	0.705		
Comorbidity (Yes vs. no)	1.219 (0.616-2.414)	0.57		
Smoking (Yes vs. no)	1.303 (0.662-2.565)	0.443		
Betel Quid (Yes vs. no)	1.334 (0.622-2.858)	0.459		
Alcohol (Yes vs. no)	1.745 (0.889-3.423)	0.105		

[A]continuous variable

Supplementary Table 5. Cross-cancer analysis of LIF mutations

Sample ID	Cancer Type	Protein Change	Mutation Type	Copy #	Start Pos
TCGA-EE-A3AC-06	Cutaneous Melanoma	W17L	Missense_Mutation		30640892
TCGA-EE-A29X-06	Cutaneous Melanoma	G22W	Missense_Mutation	gain	30640878
TCGA-AR-A0TP-01	Invasive Breast Carcinoma	C34W	Missense_Mutation	gain	30640840
TCGA-AR-A0TP-01	Breast Invasive Ductal Carcinoma	C34W	Missense_Mutation	gain	30640840
CAC_3117	Ampulla of Vater	R37H	Missense_Mutation		30640832
TCGA-HU-A4H3-01	Stomach Adenocarcinoma	N43del	In_Frame_Del		30640813
TCGA-HU-A4H3-01	Diffuse Type Stomach Adenocarcinoma	N43del	In_Frame_Del		30640813
TCGA-HU-A4H3-01	Diffuse Type Stomach Adenocarcinoma	N43del	In_Frame_Del		30640813
TCGA-BR-8485-01	Stomach Adenocarcinoma	N43K	Missense_Mutation		30640813
TCGA-BR-8485-01	Stomach Adenocarcinoma	N43K	Missense_Mutation		30640813
TCGA-BR-8485-01	Stomach Adenocarcinoma	N43K	Missense_Mutation		30640813
TCGA-AP-A1E0-01	Uterine Endometrioid Carcinoma	I48S	Missense_Mutation		30640799
coadread_dfci_2016_1231	Colorectal Adenocarcinoma	Q54*	Nonsense_Mutation		30640782
CHOL12	Intrahepatic Cholangiocarcinoma	G57C	Missense_Mutation		30640771
TCGA-DD-A4NP-01	Hepatocellular Carcinoma	G57S	Missense_Mutation	Shallow Del	30640773
TCGA-DD-A4NP-01	Hepatocellular Carcinoma	G57S	Missense_Mutation	Shallow Del	30640773
TCGA-IB-7651-01	Pancreatic Adenocarcinoma	A59V	Missense_Mutation		30640766
TCGA-IB-7651-01	Pancreatic Adenocarcinoma	A59V	Missense_Mutation		30640766
SNUC5_LARGE_INTESTINE	Mixed Cancer Types	A59V	Missense_Mutation	Gain	30640766
TCGA-XF-AAMG-01	Bladder Urothelial Carcinoma	L65F	Missense_Mutation	Gain	30640749
TCGA-XF-AAMG-01	Bladder Urothelial Carcinoma	L65F	Missense_Mutation	Gain	30640749
TCGA-FA-A7Q1-01	Diffuse Large B-Cell Lymphoma	E72G	Missense_Mutation		30640034
S01453	Small Cell Lung Cancer	E72K	Missense_Mutation		30640035
S01453	Small Cell Lung Cancer	E72K	Missense_Mutation		30640035
TCGA-E6-A2P8-01	Uterine Mixed Endometrial Carcinoma	E72Sfs*15	Frame_Shift_Del		30640035

TCGA-VS-A9UO-01	Mucinous Carcinoma	N76K	Missense_Mutation		30640021
coadread_dfci_2016_497	Colorectal Adenocarcinoma	N76Qfs*74	Frame_Shift_Ins		30640023
TCGA-50-5936-01	Lung Adenocarcinoma	N77Tfs*10	Frame_Shift_Del	Shallow Del	30640019
TCGA-50-5936-01	Lung Adenocarcinoma	N77Tfs*10	Frame_Shift_Del	Shallow Del	30640019
TCGA-50-5936-01	Lung Adenocarcinoma	N77Tfs*10	Frame_Shift_Del	Shallow Del	30640019
TCGA-50-5936-01	Lung Adenocarcinoma	N77Tfs*10	Frame_Shift_Del	Shallow Del	30640019
PANC1005_PANCREAS	Mixed Cancer Types	P84T	Missense_Mutation	Shallow Del	30639999
coadread_dfci_2016_60	Colorectal Adenocarcinoma	V86M	Missense_Mutation		30639993
587338	Colorectal Adenocarcinoma	P90L	Missense_Mutation		30639980
TCGA-CG-4460-01	Stomach Adenocarcinoma	P90L	Missense_Mutation	Shallow Del	30639980
MFE319_ENDOMETRIUM	Mixed Cancer Types	P91L	Missense_Mutation		30639977
YUDAB	Cutaneous Melanoma	P91S	Missense_Mutation		30639978
TCGA-DX-A6YT-01	Myxofibrosarcoma	A94D	Missense_Mutation	Shallow Del	30639968
TK_10	Renal Cell Carcinoma	T97M	Missense_Mutation	Shallow Del	30639959
YUISKIA	Cutaneous Melanoma	T97M	Missense_Mutation		30639959
coadread_dfci_2016_1212	Colorectal Adenocarcinoma	K99N	Missense_Mutation		30639952
TCGA-4Z-AA7Y-01	Bladder Urothelial Carcinoma	E104Q	Missense_Mutation		30639939
TCGA-4Z-AA7Y-01	Bladder Urothelial Carcinoma	E104Q	Missense_Mutation		30639939
TCGA-EE-A2GH-06	Cutaneous Melanoma	L112I	Missense_Mutation	gain	30639915
MBC-MBCProject_gjhMuoha-Tumor-SM-AXGGJ	Breast Invasive Lobular Carcinoma	N127H	Missense_Mutation	Shallow Del	30639870
TCGA-EW-A2FV-01	Breast Invasive Carcinoma, NOS	L131Sfs*36	Frame_Shift_Del	Shallow Del	30639858
BCM723T	Hepatocellular Carcinoma	A139T	Missense_Mutation		30639834
DU_145	Prostate	A141T	Missense_Mutation	Shallow Del	30639828
ESO-1096	Esophageal Adenocarcinoma	A141T	Missense_Mutation		30639828
TCGA-D1-A103-01	Uterine Endometrioid Carcinoma	A141T	Missense_Mutation		30639828
TCGA-D1-A103-01	Uterine Endometrioid Carcinoma	A141T	Missense_Mutation		30639828
TCGA-D1-A103-01	Uterine Endometrioid Carcinoma	A141T	Missense_Mutation		30639828

H080204	Hepatocellular Adenoma	L144M	Missense_Mutation		30639819
ME030	Cutaneous Melanoma	R145Q	Missense_Mutation		30639815
ME030	Melanoma	R145Q	Missense_Mutation		30639815
TCGA-CG-5733-01	Stomach Adenocarcinoma	L148del	In_Frame_Del		30639806
TCGA-CG-5733-01	Intestinal Type Stomach Adenocarcinoma	L148del	In_Frame_Del		30639806
TCGA-CG-5733-01	Stomach Adenocarcinoma	L148del	In_Frame_Del		30639806
TCGA-AN-A0FX-01	Invasive Breast Carcinoma	V151M	Missense_Mutation	gain	30639798
TCGA-AN-A0FX-01	Breast Invasive Ductal Carcinoma	V151M	Missense_Mutation	gain	30639798
TCGA-AN-A0FX-01	Breast Invasive Ductal Carcinoma	V151M	Missense_Mutation	gain	30639798
TCGA-J7-A8I2-01	Papillary Renal Cell Carcinoma	V151M	Missense_Mutation		30639798
TCGA-J7-A8I2-01	Papillary Renal Cell Carcinoma	V151M	Missense_Mutation		30639798
TCGA-AN-A0FX-01	Breast Invasive Ductal Carcinoma	V151M	Missense_Mutation	gain	30639798
TCGA-62-A46R-01	Lung Adenocarcinoma	C153Afs*14	Frame_Shift_Del		30639793
TCGA-62-A46R-01	Lung Adenocarcinoma	C153Afs*14	Frame_Shift_Del		30639793
SNU1077_ENDOMETRIUM	Mixed Cancer Types	R154C	Missense_Mutation	Shallow Del	30639789
TCGA-CV-6942-01	Head and Neck Squamous Cell Carcinoma	S157I	Missense_Mutation		30639779
TCGA-CV-6942-01	Head and Neck Squamous Cell Carcinoma	S157I	Missense_Mutation		30639779
TCGA-CV-6942-01	Head and Neck Squamous Cell Carcinoma	S157I	Missense_Mutation		30639779
MUTZ5_HAEMATOPOIETIC_AND_LYMPHOID_TISSUE	Mixed Cancer Types	Y159H	Missense_Mutation		30639774
TT2609C02_THYROID	Mixed Cancer Types	Y159H	Missense_Mutation	Shallow Del	30639774
VU1-1	Acral Melanoma	T167I	Missense_Mutation		30639749
TCGA-G4-6293-01	Colon Adenocarcinoma	T172S	Missense_Mutation		30639735
TCGA-BS-A0UF-01	Uterine Endometrioid Carcinoma	S173L	Missense_Mutation		30639731
TCGA-FS-A1ZB-06	Cutaneous Melanoma	S173L	Missense_Mutation		30639731
TCGA-BS-A0UF-01	Uterine Endometrioid Carcinoma	S173L	Missense_Mutation		30639731
TCGA-FS-A1ZB-06	Cutaneous Melanoma	S173L	Missense_Mutation		30639731

TCGA-BS-A0UF-01	Uterine Endometrioid Carcinoma	S173L	Missense_Mutation		30639731
RL952_ENDOMETRIUM	Mixed Cancer Types	S173L	Missense_Mutation		30639731
TCGA-90-A4ED-01	Lung Squamous Cell Carcinoma	D176N	Missense_Mutation	Shallow Del	30639723
TCGA-90-A4ED-01	Lung Squamous Cell Carcinoma	D176N	Missense_Mutation	Shallow Del	30639723
TCGA-CQ-6221-01	Head and Neck Squamous Cell Carcinoma	K181N	Missense_Mutation	gain	30639706
TCGA-CQ-6221-01	Head and Neck Squamous Cell Carcinoma	K181N	Missense_Mutation	gain	30639706
TCGA-KP-A3W1-01	Uterine Serous Carcinoma/Uterine Papillary Serous Carcinoma	K181_K190del	In_Frame_Del	gain	30639679
TCGA-G9-6342-01	Prostate Adenocarcinoma	G189W	Missense_Mutation		30639684
TCGA-A5-A0G2-01	Uterine Serous Carcinoma/Uterine Papillary Serous Carcinoma	K192N	Missense_Mutation		30639673
coadread_dfci_2016_112	Colorectal Adenocarcinoma	Q200K	Missense_Mutation		30639651
TCGA-D1-A17Q-01	Uterine Endometrioid Carcinoma	A201T	Missense_Mutation		30639648
TCGA-D1-A17Q-01	Uterine Endometrioid Carcinoma	A201T	Missense_Mutation		30639648
TCGA-D1-A17Q-01	Uterine Endometrioid Carcinoma	A201T	Missense_Mutation		30639648

Supplementary Methods

LIF library preparation

LIF sequencing was performed using the GeneRead DNAseq Custom Panel V2 (QIAGEN, Hilden, Germany), which amplifies 74 amplicons covering 8.4 kb, containing intron, exon and 2K of 5' upstream of LIF sequence. The multiplex PCRs were carried out using 10 ng of genomic DNA as templates for each primer mix pool. In total, 40 ng of genomic DNA were used for each sample. The PCR amplification were performed using 24 PCR cycles for frozen tissues and matched normal, and 25 PCR cycles for the formalin-fixed paraffin-embedded (FFPE) samples. The 4-pool PCR products for each sample were pooled and purified according to manufacturer’s protocol, and were applied to Illumina TruSeq Nano DNA Sample Prep Kit (Illumina, San Diego, CA, USA). The purified 100 ng pooled PCR product were used as input for end-repaired, adenylation, adapter ligation and enrichment of DNA fragments according to manufacturer’s protocol. The libraries were quantified using a Qubit dsDNA HS Assay Kit (Life Technologies, MA, USA) and HT DNA High Sensitivity LabChip Kit (Caliper Life Sciences GmbH, Mainz, Germany).

Illumina MiSeq sequencing and data analysis

The libraries were diluted to a final concentration of 4nM and pooled. Both pooled

library and PhiX control (Illumina, San Diego, CA, USA) were denatured by 0.2 N NaOH and diluted to 20 pM. The 15% denatured PhiX was added to the pooled library. The mixture of Phix and pooled library was diluted to a final concentration of 12 pM, and then applied to the MiSeq reagent cartridge (Illumina, San Diego, CA, USA) according to the manufacturer´s instructions. The deep-sequencing was performed using MiSeq Reagent Kits v2 (Illumina, San Diego, CA, USA) following a paired-end sequencing (2 × 150 bp) protocol. The sequencing data were processed by MiSeq Reporter (MSR; Illumina, San Diego, CA, USA; v2.5.1.3), which uses BWA [ref] (v0.6.1-r104-tpx) and Somatic Variant Caller (Illumina, San Diego, CA, USA; v3.5.2.1.) for sequence alignment and variant calling. The called variants were annotated by Variant Studio (Illumina, San Diego, CA, USA). To identify the germline variants, we sequenced tumors and matched normal samples in parallel. However, in tumor-only samples, we regarded variants which are registered in polymorphisms databases as germline. Allele frequencies threshold of germline variants were set at >20%. The somatic mutations in tumors with matched normal were identified by subtracting germline alterations. Owing to lacking the definite identification of germline variants in tumor-only sequencing, we excluded the variants identified in polymorphisms databases. To avoid sequence error caused by DNA modification arising during FFPE samples storage, the somatic variant calling

threshold was set at 5% and 10% for variants with ≥ 1000× and ≥ 100×, respectively.

Sanger sequencing

Genomic DNA from FFPE and frozen tumors were performed for mutation detection using Sanger sequencing. The primers were designed to amplify the region of exon 1 and 2 which contain the sequence of signal peptide. The sequences of primer were as follows: exon 1 forward primer, 5’-CCCAAGTGTTCGTGTGTCT-3’ and 5’-CCCATTTGAGCATGAACCT-3’; exon 2 forward primer, 5’-GTCAGTATCCCAGGGGTAACTTA-3’ and reverse, 5’-TCATGGCTTCTTCCTGACT-3’. The both forward primers of exon 1 and 2 were selected for sequencing primer.

Reagents

The reagents used included recombinant leukemia inhibitor factor (Sigma, L5283), AZD0530 (LC Laboratories, S8906), Collagen (Advanced BioMatrix, 5005-B) and Lipofectamine RNAiMAX transfection reagent (Invitrogen, 13778150).

Detection of LIF concentration in culture supernatant of cancer cells

The secreted LIF levels were determined using a bead-based cytokine assay (Bio-Rad Laboratories, Hercules, CA). Standard curves for LIF was generated using the

reference LIF concentrations provided by the manufacturer. Cultural supernatants derived from cancer cells were harvested after 48 hours incubation.

Wound-healing assay

Cell migration patterns were examined with the 2D wound healing assay. Cancer cells were seeded at a density of 5×10^5 cells/3.5 cm culture dish and grown for three days to confluence. Cell layers were carefully wounded with sterile 1000 µl pipette tips. Wounded cell layers were washed five times with PBS, replenished with complete medium, and placed in a microscopic incubator for time-lapse experiments under a 10X phase contrast objective (Olympus IX83). Each experiment was performed at least three times.

Cell cultures

The NPC-derived cancer cell line, BM1 [1], and BM1-derived cell strains (LIFc and $LIF^{+/-}$) were cultured in RPMI1640 supplemented with 10% fetal bovine serum (Invitrogen). BM1 cells were authenticated via 16 core short tandem repeat (STR) locus profiling (analyzed by Bioresource Collection and Research Center, Taiwan). Primary human umbilical vein endothelial cells (HUVECs) were purchased from the Bioresource Collection and Research Center (Taiwan) and grown in EGM™-

2 medium (Lonza).

Establishment of LifeAct_RFP or LifeAct_GFP2-expressing cell pools

For visualization of the actin cytoskeleton in live-cell imaging, cancer or undifferentiated HUVEC cells were infected with adenovirus containing either LifeAct-RFP or LifeAct-GFP2 (Ibidi) at a pre-optimized multiplicity of infection (MOI) according to the manufacturer's protocol. For selection of stable clones, puromycin (2 μg/ml) (Invitrogen) was added to culture medium at 48 h post-infection. Cells stably expressing LifeAct-RFP or LifeAct-GFP2 were confirmed via microscopic examination.

Live-Cell Imaging

Time-lapse experiments were performed in a chamber maintained at 37℃ and 5% CO_2 under a phase contrast inverted microscope (Olympus IX83). Simultaneous acquisition of two-color fluorescent images was achieved every 5 min and movies generated using the CellSens imaging software (Olympus).

Immunofluorescence

Cells were grown on sterile glass coverslips or chamber slides (Nunc) and fixed

with 4% paraformaldehyde, followed by permeabilization with 0.3 % Triton X-100 and blocking with 8% normal goat serum. Cells were subjected to immunostaining using primary antibodies and visualized with Alexa Fluor-conjugated secondary antibodies (Invitrogen). Nuclei were stained with Hoechst 33342 (Invitrogen).

Transfection

For small interfering RNA transfection, cells were seeded in a 6-well culture plate at a density of 2×10^5 cells/well for 24 h prior to transfection. Cells were transfected with SMARTpool double strand small interfering RNA (siRNA) against LIFR (target sequence: CGGAAACGAGAAUGGAUUA, GAGAGUAACAACACGGGAA, GCAAUAUCUAGCAGCGUUA, AGAACAAACCAAACGAUUA, Thermo Scientific) or one pair of following siRNAs targeting YAP1 (target sequences: 5'-CCUCACAGCAGAACCGUUUtt-3', 3'-ttGGAGUGUCGUCUUGGCAAA-5', 5'-GGUCAGAGAUACUUCUUAAtt-3', 3'-ttCCAGUCUCUAUGAAGAAUU-5', 5'-CCUUAACAGUGGCACCUAUtt-3', 3'-ttGGAAUUGUCACCGUGGAUA-5', 5'-GCUGCCACCAAGCUAGAUAtt-3', 3'-ttCGACGGUGGUUCGAUCUAU-5', MDBio, Inc.) using Lipofectamine RNAiMAX transfection reagent to achieve a final concentration of 50 nM. A non-target siRNA (Thermo Scientific) was used as the negative control. For the HUVEC displacement assay, cells were collected at 24 h

post-transfection. For western blot analysis, protein lysates were harvested at 48 h post-transfection.

Supplementary reference

1. Liao SK, Perng YP, Shen YC, Chung PJ, Chang YS, Wang CH. Chromosomal abnormalities of a new nasopharyngeal carcinoma cell line (NPC-BM1) derived from a bone marrow metastatic lesion. *Cancer Genet Cytogenet* **103**, 52-58 (1998).
