# Supplementary material for: Cytoplasmic LIF reprograms invasive mode to enhance NPC dissemination through modulating YAP1-FAK/PXN signaling
Source: Nat Commun. 2018 Nov 30;9:5105. doi: 10.1038/s41467-018-07660-6 (PMC6269507; doi:10.1038/s41467-018-07660-6)
Supplement: Supplementary file 3 — Description of Additional Supplementary Files [file 41467_2018_7660_MOESM3_ESM.pdf]

Title: Supplementary Movie 1
Description: Migration pattern of WT cells in wound-healing assay.

Title: Supplementary Movie 2
Description: Migration pattern of cLIF cells in wound-healing assay.

Title: Supplementary Movie 3
Description: Migration pattern of LIF+/- cells in wound-healing assay.

Title: Supplementary Movie 4
Description: WT cell-induced HUVEC layer damage.

Title: Supplementary Movie 5
Description: cLIF cell-induced HUVEC layer damage.

Title: Supplementary Movie 6
Description: LIF+/- cell-induced HUVEC layer damage.
