# Supplementary material for: Author Correction: Cytoplasmic LIF reprograms invasive mode to enhance NPC dissemination through modulating YAP1-FAK/PXN signaling
Source: Nat Commun. 2026 Nov 4;17:11416. doi: 10.1038/s41467-026-79112-5 (PMC13736888; doi:10.1038/s41467-026-79112-5)
Supplement: Supplementary file 1 [file 41467_2026_79112_MOESM1_ESM.pdf]

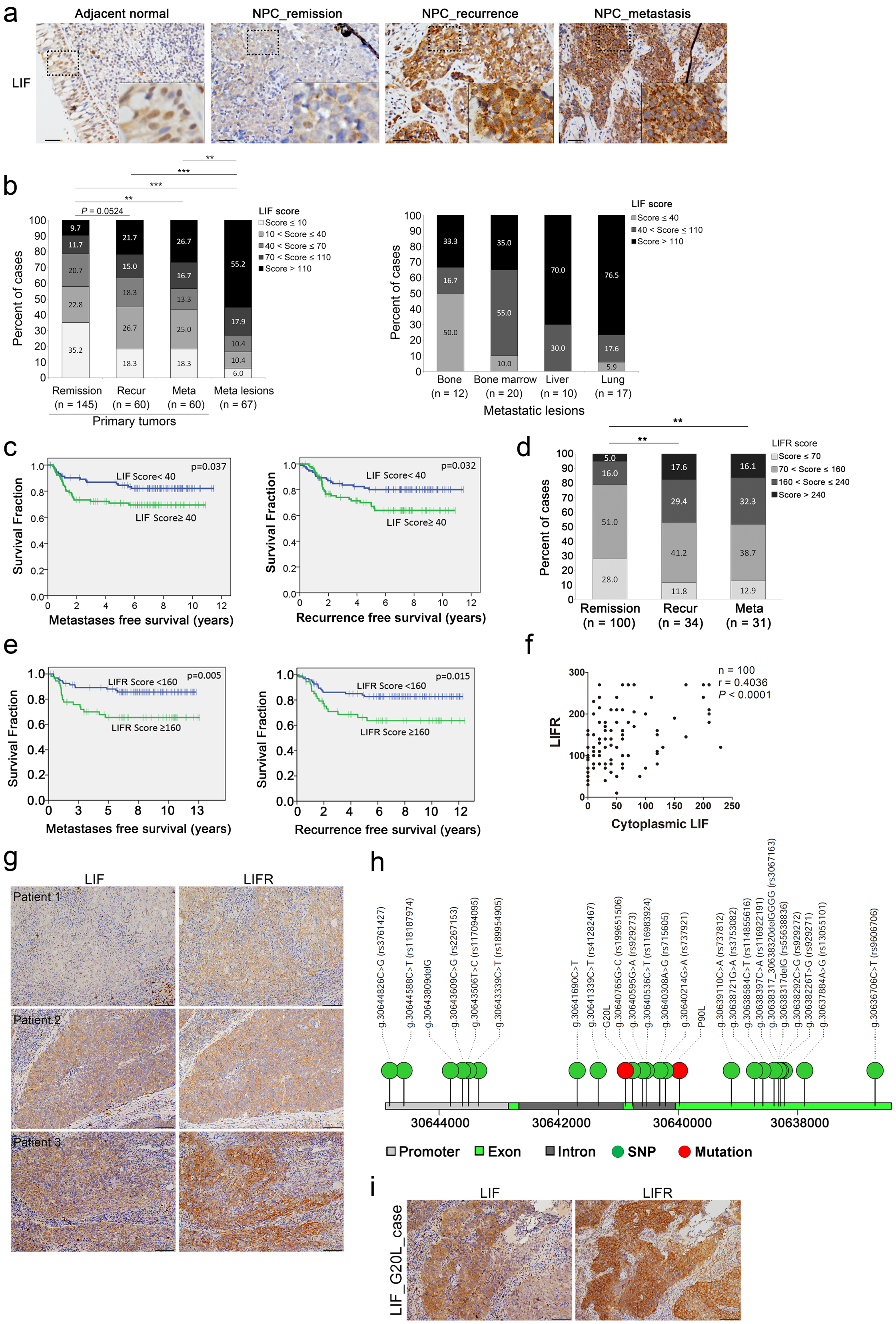

a
Adjacent normal
NPC_remission
NPC_recurrence
NPC_metastasis
LIF
b
LIF score
Score ≤ 10
10 < Score ≤ 40
40 < Score ≤ 70
70 < Score ≤ 110
Score > 110
P = 0.0524
**
***
***
**
Percent of cases
Remission (n = 145)
Recur (n = 60)
Meta (n = 60)
Meta lesions (n = 67)
Primary tumors
35.2 22.8 20.7 11.7 9.7
18.3 26.7 18.3 15.0 21.7
18.3 25.0 13.3 16.7 26.7
6.0 10.4 10.4 17.9 55.2
LIF score
Score ≤ 40
40 < Score ≤ 110
Score > 110
Percent of cases
50.0 16.7 33.3
10.0 55.0 35.0
30.0 70.0
5.9 17.6 76.5
Bone (n = 12)
Bone marrow (n = 20)
Liver (n = 10)
Lung (n = 17)
Metastatic lesions
c
Survival Fraction
LIF Score< 40
LIF Score≥ 40
p=0.037
Metastases free survival (years)
p=0.032
Recurrence free survival (years)
d
LIFR score
Score ≤ 70
70 < Score ≤ 160
160 < Socre ≤ 240
Score > 240
Percent of cases
28.0 51.0 16.0 5.0
11.8 41.2 29.4 17.6
12.9 38.7 32.3 16.1
Remission (n = 100)
Recur (n = 34)
Meta (n = 31)
**
**
e
Survival Fraction
LIFR Score <160
LIFR Score ≥160
p=0.005
Metastases free survival (years)
p=0.015
Recurrence free survival (years)
f
n = 100
r = 0.4036
P < 0.0001
LIFR
Cytoplasmic LIF
g
LIF
LIFR
Patient 1
Patient 2
Patient 3
h
g.30644826C>G (rs3761427)
g.30644588C>T (rs118187974)
g.30643809delG
g.30643609C>G (rs2267153)
g.30643506T>C (rs117094095)
g.30643339C>T (rs189954905)
g.30641690C>T
g.30641339C>T (rs41282467)
G20L
g.30640765G>C (rs199651506)
g.30640595G>A (rs929273)
g.30640536C>T (rs116983924)
g.30640308A>G (rs715605)
g.30640214G>A (rs737921)
P90L
g.30639110C>A (rs737812)
g.30638721G>A (rs3753082)
g.30638584C>T (rs114855616)
g.30638397C>A (rs116922191)
g.30638317_30638320delGGGG (rs3067163)
g.30638317delG (rs55638836)
g.30638292C>G (rs929272)
g.30638226T>G (rs929271)
g.30637884A>G (rs13055101)
g.30636706C>T (rs9606706)
30644000
30642000
30640000
30638000
Promoter
Exon
Intron
SNP
Mutation
i
LIF
LIFR
LIF_G20L_case
